# Abernethy malformation: a case report

**DOI:** 10.1186/1471-2431-12-57

**Published:** 2012-05-29

**Authors:** Ashish Pathak, Nitin Agarwal, Jagdish Mandliya, Prateek Gehlot, Mamta Dhaneria

**Affiliations:** 1 Department of Pediatrics, R.D. Gardi Medical College, Ujjain, India; 2 Division of Global Health, IHCAR, Karolinska Institutet, Stockholm, Sweden; 3 Department of Pediatric Surgery, R.D. Gardi Medical College, Ujjain, India; 4 Department of Radiology, R.D. Gardi Medical College, Ujjain, India

**Keywords:** Abernethy malformation, Ultra-sonography, Associations, Complications, India

## Abstract

**Background:**

Abernethy malformation is a very rare congenital vascular malformation defined by diversion of portal blood away from liver. It is commonly associated with multiple congenital anomalies. We present a case of Abernethy malformation, without associated congenital anomalies from India.

**Case presentation:**

A 5-year-old female child presented with short history of jaundice. A provisional diagnosis of acute viral hepatitis was made in view of clinical presentation and local endemicity of viral hepatitis A. Persistence of jaundice on follow up after 4 weeks led to detailed investigations. Ultrasound and doppler study of abdomen revealed drainage of portal vein into inferior vena cava. CT angiography was performed which confirmed the diagnosis of Type 1 b Abernethy malformation without associated major anomalies. We discuss the common clinical presentations, associated anomalies, diagnostic workup and treatment options of this disorder.

**Conclusion:**

The treatment of the patients with congenital porto-systemic shunts depends on the site of the shunt, associated congenital anomalies and the extent of liver damage but the prognosis depends on the complications irrespective of anatomical type. However, the extent of associated abnormalities should not deter paediatricians to refer patients for treatment. Whenever possible closure of the shunt should be advised for cure or to prevent complications. Only symptomatic type I patients with absence of possibility to close the shunt may require liver transplant. Long-term follow-up is indicated for all patients.

## Background

John Abernethy first reported congenital absence of portal vein and congenital mesenterico-caval shunt in 1793 [1]. Abernethy malformation is defined as congenital diversion of portal blood away from the liver by either end-to-side or side-to-side shunt [2]. Morgan and Superina [3] classified congenital extrahepatic portosystemic shunt (CEPSh) into two types (See Table 1). Till the year 2008 only forty cases of CEPSh type I and twenty-two of CEPSh type II, had been described in literature [4,5]. This is despite the tremendous advances in the diagnostic medical imaging, thus underpinning the rarity of these cases. Only one paediatric case has been reported from India [6]. In this article we describe a patient diagnosed to have type I b Abernethy malformation after prolonged jaundice.

**Table 1:** **Morgen and Superina**[3]**classification of congenital extra hepatic portosystemic shunt**

**Extra hepatic portosystemic shunts**	
Type I	Absence of intrahepatic portal veins
Type I a	Superior mesenteric and splenic vein drain separately
	into inferior vena cava
Type I b	Superior mesenteric vein and splenic vein form a common
	Trunk before draining into the inferior vena cava
Type II	Important collateral, patent intrahepatic veins

## Case presentation

We present a case of 5 year old girl who presented to paediatric outpatient clinic of R.D. Gardi Medical College and C.R. Gardi Hospital, Surasa, Ujjain, India with complains of decreased appetite since past four to five days, yellowish discoloration of sclera noticed since past two days and deep yellow coloured urine since past two days. Patient had no history of clay coloured stools, fever, vomiting, haematemesis, malena or history suggestive of hepatic encephalopathy. The perinatal history was uneventful with no history of umbilical sepsis. The child had no significant past medical history including that of jaundice. Immunization of the child was appropriate for age. At initial examination her anthropometric measurements showed a weight of 15 Kg and a height of 110 cm. The weight-for-age was between 3^rd^ to 15^th^ percentiles; height-for-age between 50^th^ to 85^th^ percentiles and weight-for-height below 3^rd^ percentile, according to the WHO Reference Growth Standards 2007 (http://www.who.int/childgrowth/standard/chart_catalogue ). The anthropometric findings were suggestive of acute on chronic malnutrition with stunting. The dietary evaluation revealed a diet inadequate in calories (by one third), proteins (by one fourth) and iron (by half) for her age and sex. The vitals at presentation were heart rate of 120 per minute, respiratory rate of 20 per minute and blood pressure of 98/60 mm of Hg in right in right upper limb in supine position. Four-limb blood pressure measurement was also normal. Icterus was noticed. Examination of ears, nose and throat showed no abnormality. No skin rash was present. No clubbing, cyanosis or oedema of arms or legs was noted. Cardiovascular examination was normal. The lungs were clear on percussion and auscultation. The abdomen was soft with no organomegaly or clinical ascites. A provisional diagnosis of acute viral hepatitis was made based on history, clinical examination and local endemicity of acute viral hepatitis caused by hepatitis A or hepatitis E. Only blood investigation advised was serum bilirubin, which showed total bilirubin 9.29 mg/dl with direct bilirubin of 7.38 mg/dl and indirect bilirubin of 2.41 mg/dl. Patient was treated symptomatically for viral hepatitis and asked to follow up if any danger signs of acute viral hepatitis appear or after 4 weeks. No serological confirmation of viral hepatitis was deemed necessary in view of acute viral hepatitis caused by hepatitis A virus being an endemic disease in the geographic region.

**Figure 1:**
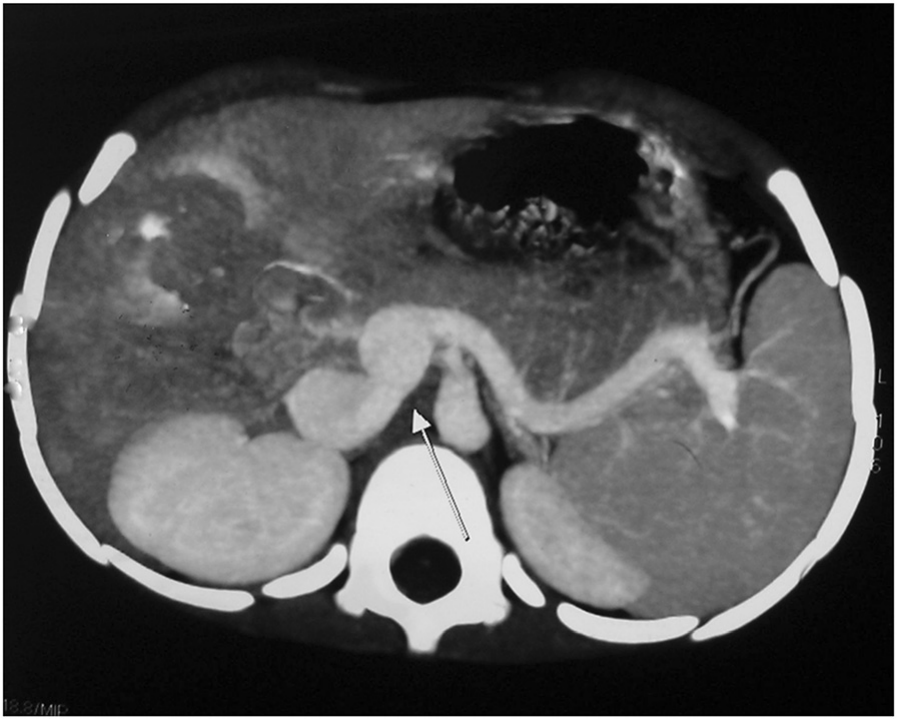
**Contrast enhanced CT scan of upper abdomen with MIP to include of whole thickness of portal vein shows aberrant opening of portal vein into inferior vena cava (marked by white solid arrow in the figure).** Note dilated inferior vena cava cranial to confluence in the image.

**Figure 2:**
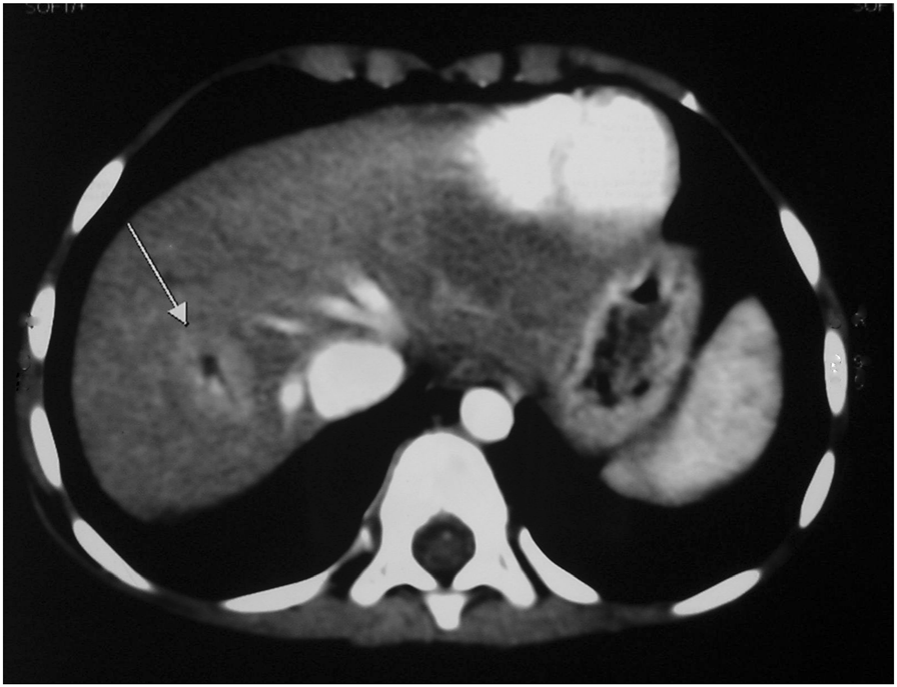
CT scan abdomen: Mass lesion in right lobe of liver (marked by white solid arrow).

Patient followed four weeks later with persistence of yellowish discoloration of sclera but had no other symptoms. Her clinical examination was unremarkable. A liver profile, complete blood counts and abdominal ultrasonography (USG) were ordered. The complete blood counts were within normal limits and did not reveal any evidence of hypersplenism. The liver profile showed total bilirubin 3.4 mg/dl with direct bilirubin of 3.23 mg/dl and indirect bilirubin of 0.22 mg/dl; aspartate transaminase (AST) 98.2 IU/L (normal values 15–46 IU/L); alanine transaminase (ALT) 109.3 IU/L (normal values 13–69 IU/L); alkaline phosphatase 269.3(normal values 38–126 IU/L); prothombin time (PT) 14 with control of 16; INR 1.2. Abdominal ultrasound showed course ecotexture of the liver with anomalous portal vein. The size of portal vein was 8 mm. The portal vein drained into inferior vena cava. A CT angiography was advised following the above abdominal ultrasound report. The contrast enhanced CT scan of upper abdomen with MIP to include of whole thickness of portal vein revealed that the superior mesenteric vein and the splenic vein join to form a confluence that drained directly in the inferior vena cava. The portal vein drained in the inferior vena cava at the level of head of pancreas caudal to the intrahepatic portion (Figure 1 and 2). A dilated inferior vena cava is seen in Figure 2. The CT scan also showed a mass lesion in right lobe of liver probably an adenoma or focal nodular hyperplasia (Figure 2). In view of the above features and absence of portal vein radicals in liver parenchyma a diagnosis of Abernethy malformation type 1 b was made. To complete the work up a fasting blood sugar and serum ammonia was done which were normal. (Blood sugar 71 mg/L and serum ammonia 20 μgm/dL (normal values 10–80 μgm/dL). A skeletal survey did not show any skeletal abnormalities. An echocardiogram with colour doppler did not show any associated congenital cardiac defects.

## Consent

Written informed consent was obtained from the patient’s father for publication of this case report and accompanying images.

## Discussion

Due to the endemicity of acute viral hepatitis caused by hepatitis A and hepatitis E most clinicians do not investigate a typical “viral hepatitis” case. In the present case the jaundice can be explained only by the viral hepatitis alone. But persistence of jaundice led us to investigate the present case. Thus, the possibility of missing a diagnosis in resource poor setting is high where both the diagnostic facilities and follow up of patients is often inadequate. However, one more basic indicator pointing towards a chronic disease were the anthropometric measurements. The importance of diagnostic imaging and follow-up of disease course is illustrated in this case. We now discuss various possible clinical presentations and associated congenital anomalies of the Abernethy malformations followed by diagnostic workup and treatment options.

Congenital intra-hepatic portosystemic shunts can present in the early neonatal period with growth restriction [7], galactosemia [8-10], neonatal cholestasis [11], and hepatic encephalopathy [12]. Cases of CEPSh have a variable presentation. In countries having neonatal screening programmes, some cases can be diagnosed by neonatal screening tests due to presence of galactosemia [8-10]. Many patients are diagnosed due to associated defects like heart disease, which are present in up-to 60% of the patients [4,12-14]. Symptoms of secondary complications like hypoglycemia, hyperammonemia, encephalopathy and cardiac failure can be transient and can resolve spontaneously [12]. Subclinical course is more common and some patients might not have any symptoms throughout life [12,13,15]. Later, other lesions like nodular regenerative hyperplasia, partial nodular transformation, hepatoblastoma, hepatocellular carcinoma and adenoma can develop [4].

The two possible explanations of development of these neoplasm are: a) diversion of hepatotrophic substances like insulin and glucagon away from liver leading to altered development, function and regenerative capacity of the liver and b) increased arterial hepatic flow [4]. Our patient does not have any evidence of hepatic neoplasm (normal alfa feto protein) at present after more than two year follow up, however in view of the documented increased risk of hepatocellular carcinoma and adenoma we have advised a regular radiological and serological surveillance at six monthly interval.

Despite presence of porto-systemic shunt, patients with CEPSh usually do not present with encephalopathy. Hyperammonemia may be present without encephalopathy especially at younger age. Clinical encephalopathy is more common at older age. The possible explanations are a) an increased sensitivity of the aging brain to toxic effects of ammonia and other toxic metabolites b) extent of shunt determined by the portal/systemic shunt ratio. A shunt ratio of more than 60% may predict the age of onset of hepatic encephalopathy [6,16]. In our case, the ammonia concentration was within normal range and patient has had no clinical evidence of encephalopathy till date.

Varying degree of dyspnoea on exertion could be a presentation of portopulmonary hypertension or hepatopulmonary syndrome. Hepatopulmonary syndrome presents with hypoxemia and or orthopnea with clubbing; due to abnormal intrapulmonary vascular dilatations [13,17]. In our patient there is no evidence of hepatopulmonary syndrome, till date clinically as well as on echocardiography.

Congenital hepatic shunt can also present with hypoglycaemia. This might be due to combined effect of defective uptake of glucose and hyperinsulinemia due to reduced hepatic degradation of normal quantity of secreted insulin [18]. A work up of common associated anomalies was done but revealed no anomaly. These include cardiac defects (60%), biliary atresia (20%), polysplenia (20%), situs inversus (10%) and malrotation (10%) [14,19].

A diagnosis of Abernethy malformation is made by non-invasive cross-sectional imaging techniques such as ultrasound, CT or MRI, which show the shunt and any intrahepatic portal vein branches [17].

The planning of treatment is dependent on the type of shunt as classified by Morgan and Superina [3] and needs to be tailored to the individual patient in accordance with preoperative evaluation [15]. Usually in patients with CEPSh type I, occlusion of shunt is not performed, as it is the only drainage route for the mesenteric venous blood. But recently published experience by several authors [15,17] point that many patients with CEPSh type I malformations might have small portal vein radicals which cannot be seen on ultrasonography but could be visualized on shunt angiography. The balloon occlusion test of the fistula can also be done [15,17]. This test helps to decide on a single stage or a two-staged shunt closure procedure. A two-step procedure allows the portal branches to enlarge slowly and can avoid acute severe portal hypertension [15,17]. Extremely hypoplastic or undetectable portal veins will require banding of the fistula before closure [17]. Shunt closure results in restoration of intrahepatic portal flow in most patients [17]. Clinical improvement in form of regression of benign liver masses, and regression or stabilization of pulmonary, cardiac, neurological, and renal complications is seen in patients post shunt [17]. CEPSh typeI patients also need clinical, biochemical and imaging follow-up [17]; as is done in our patient. For patients with CEPSh type I developing complications like encephalopathy or neoplasms, till recently liver transplant was the only treatment option [6,15,19,20], but transplant should be reserved for exceptionally complex anatomy where closure of the shunt is not possible.

Patients with CEPSh type II with hepatic encephalopathy can benefit by early shunt occlusion surgery [15,17]. Reconstruction of the portal vein should be done early to avoid mesenteric venous congestion [20]. Shunt surgery when possible is the treatment of choice for CEPSh type I and type II. Our patient is asymptomatic at present but since the patient has nodular lesion in the liver she should be considered “symptomatic”. In view of this our patient has been counselled for need for shunt surgery.

## Conclusions

Persistence of jaundice should be investigated at all ages. Ultrasound is useful tool for screening of congenital anomalies associated with liver. In resource poor settings the diagnosis of portosystemic shunts can be missed or significantly delayed due to lack of resources need for the diagnosis, including availability of trained ultra-sonologists. The prognosis of the patients with congenital portosystemic shunts depends on the site of the shunt as determined by Morgan and Superina classification, the associated congenital anomalies and the extent of liver disease. Many patients will benefit from shunt surgery. The extent of associated abnormalities should not deter paediatricians to refer patients for treatment. A long-term follow-up is indicated for all asymptomatic patients of Abernethy malformation.

## Competing interests

The authors declare that they have no competing interests.

## Authors’ contributions

AP, NA and JM collected the clinical details and photographs of this case report. AP performed the literature review, designed and drafted the manuscript. PG helped in interpretation of radiological data and selection of photographs. MD verified the diagnosis and other scientific facts. All the authors are responsible for clinical follow up of the case. All authors read and approved the final manuscript.

## Pre-publication history

The pre-publication history for this paper can be accessed here:

http://www.biomedcentral.com/1471-2431/12/57/prepub
